# Endometriosis: A concise practical guide to current diagnosis and treatment

**DOI:** 10.4274/jtgga.2018.0026

**Published:** 2018-08-06

**Authors:** İbrahim Alkatout, Ivo Meinhold-Heerlein, Jörg Keckstein, Liselotte Mettler

**Affiliations:** 1 Department of Gynecology and Obstetrics, Kiel School of Gynecological Endoscopy, University Hospitals Schleswig-Holstein, Kiel, Germany; 2 Department of Obstetrics and Gynecology, RWTH Aachen University Faculty of Medicine, Aachen, Germany; 3 Department of Obstetrics and Gynecology, Certified Clinical-Scientific Level III Center for State Hospital of Villach, Carinthia, Austria

**Keywords:** Endometriosis, laparoscopy, robotic surgery, pain, infertility

## Abstract

This book addresses the management of endometriosis from a holistic approach, including theoretical principles, conservative treatment of the condition, diagnostic and surgical procedures, as well as the outcome of research. Endometriosis and its treatment are complex issues. We believe that a combination of treatment modalities is needed in order to effectively address the pain, infertility, and other difficulties associated with endometriosis. In addition to theory and scientific principles, minimally invasive surgical procedures for the treatment of endometriosis are described in a stepwise manner. Separate sections of the books are devoted to specific conditions, urological procedures, and general surgical procedures because the condition is ideally treated on an interdisciplinary basis. Areas of overlap with other specialties are also addressed.

## Introduction

Despite a large number of reports on endometriosis, a practical guide for endoscopic surgical treatment does not exist. The integration of traditional procedures with alternative methods of treatment has also not been addressed satisfactorily. To resolve this problem, the editors of the present book requested a group of expert researchers and physicians to describe their view of the management of endometriosis (1). A team of dedicated surgeons wrote separate chapters on the existing surgical approaches for the treatment of the condition. 

In view of the fact that endometriosis is a benign but chronic estrogen-dependent disease and a lifelong problem for the patient, its early identification and appropriate treatment are essential. In addition to traditional theories concerning the pathogenesis of various types of endometriosis, the recent published literature suggests that a variety of additional factors play a role in the development and spread of the disease, such as genetic predisposition, an abnormal peritoneal environment, stem cells, immune dysfunction, and inflammation (2,3). A number of therapies have been suggested and are, in part, effective. However, surgery - usually endoscopic surgery - still is the principal procedure for the diagnosis and treatment of endometriosis. Therefore, the present work focuses on the individual steps of surgical procedures. 

The purpose of the book is two-fold: a) improve the patient’s comprehension of the problems that may arise and are resolved during surgery; b) guide surgeons in performing the appropriate surgical procedure in an efficient manner (4,5). All forms of endometriosis must be managed by surgery in order to treat the condition effectively. 

It should be noted that the majority of treatments currently used for endometriosis are based on surgery. The large majority of medical therapies rely on manipulating ovarian steroid hormones, but fail to achieve a complete response in every patient. The authors of this book hope that continued research will disclose new perspectives for the development of novel treatment strategies (2,6). The extensive support from the industry in terms of technology, instruments, optical aids, and healthcare has made endoscopic surgery safer, simpler, less invasive, and decidedly more effective. 

## Outline of the book

The book addresses theoretical principles, clinical experience, and scientific conclusions. A variety of surgical techniques for different manifestations of endometriosis are discussed; illustrations and tables are provided. This work serves as a reliable guide for beginners as well as experts. The above mentioned aspects - background, diagnosis, and treatment options for endometriosis - are divided as follows:

1. Theoretical Principles, Research Results and Conservative Treatment

2. Diagnostic Workup and Preparation for Surgery

3. Surgical Procedures for Various Manifestations of Endometriosis

4. Specific Situations

5. Urological Approaches

6. General Surgical Approach

Gynecologists concerned with conservative treatment and surgery will be able to widen their perspectives. This book should enhance their ability to provide state-of-the-art diagnostic investigation and treatment strategies for endometriosis.

The historical background is followed by the etiology, pathogenesis, and pathology of endometriosis. The anatomy of the region, and the reasons for radical treatment are addressed. These sections are followed by internationally accepted classification systems. One chapter addresses medical treatments and the currently undervalued measures of rehabilitation and appropriate nutrition.

The book offers a complete overview of diagnostic investigation and surgical techniques for various forms of endometriosis. The approaches include conventional laparoscopic and robot-assisted approaches, as well as urologic and general surgical procedures (7). The management of endometriosis in patients with infertility is also addressed in detail.

This work has been produced by an Austrian-German group of scholars, associates, and experts at the Kiel School of Gynecological Endoscopy. The latter is a gynecologic training center located at the department of obstetrics and gynecology at Campus Kiel, which is one of the University Hospitals of Schleswig-Holstein in Germany.

One of the foremost aims of the book was to enhance and promote effective cooperation between patients, medical experts, researchers, as well as producers and developers of medical technology (8). This was the reason for publishing the book through Endo Press GmbH, Tuttlingen, Germany, and the KARL STORZ Company. Thanks to these publishers, the book can be downloaded free of cost from the Karl Storz media library.

## Highlights

This book mainly addresses the actual procedure of endoscopic surgery, based on conventional laparoscopic and robotic-assisted techniques. Procedures performed within the anatomical scope of gynecology, as well as urologic and general surgical procedures are included. 

The chapter on the origin of the disease summarizes currently-known aspects of the pathogenesis of endometriosis, with specific attention given to the mechanisms underlying the vascularization of lesions and the role of immune factors. Furthermore, the importance of hormones, immune cells, and cytokine signaling is addressed. Current pharmaceutical options for the management of pain in women with this persistent but potentially manageable disease are described. Internationally known and accepted classifications of the disease are highlighted. Fertility treatment and endometriosis are also given attention (9,10).

## Evaluation of the book

This book encompasses a large number of overlapping medical specialties, as such the editors regard it as a comprehensive answer to the need for interdisciplinary treatment of endometriosis. Specialists of visceral surgery, urology, and internal medicine will be able to identify the interdisciplinary aspects of the condition and their treatment. 

In view of the currently extensive communication among the various specialties of medicine, we invited a number of leading surgeons, scientists, and teachers from all over the world to contribute to this book. It presents the newest treatment strategies for endometriosis. Furthermore, the open access e-Book version meets the global demands of unlimited exchange and is freely available online to the international community. Last but not least, this textbook aims to improve the standard of healthcare for women.
